# A-674563, a putative AKT1 inhibitor that also suppresses CDK2 activity, inhibits human NSCLC cell growth more effectively than the pan-AKT inhibitor, MK-2206

**DOI:** 10.1371/journal.pone.0193344

**Published:** 2018-02-22

**Authors:** Paige M. Chorner, Roger A. Moorehead

**Affiliations:** Department of Biomedical Sciences, Ontario Veterinary College, University of Guelph, Guelph, Ontario, Canada; University of South Alabama Mitchell Cancer Institute, UNITED STATES

## Abstract

AKT is a serine-threonine kinase implicated in tumorigenesis as a central regulator of cellular growth, proliferation, survival, and metabolism. Activated AKT is commonly overexpressed in non-small cell lung cancer (NSCLC) and accordingly AKT inhibitors are under clinical investigation for NSCLC treatment. Thus far, the AKT inhibitors being evaluated broadly target all three (1–3) AKT isoforms but recent evidence suggests opposing roles in lung tumorigenesis where loss of *Akt1* inhibits while the loss of *Akt2* enhances lung tumor development. Based on these findings, we hypothesized that selective inhibition of AKT-1 would be a more effective therapeutic strategy than pan-AKT inhibition for NSCLC treatment. Using six NSCLC cell lines, we found that the AKT-1 inhibitor, A-674563, was significantly more effective at reducing NSCLC cell survival relative to the pan-AKT inhibitor MK-2206. Comparison of the downstream effects of the inhibitors suggests that altered cell cycle progression and off-target CDK2 inhibition are likely vital to the improved efficacy of A-674563 over MK-2206.

## Introduction

Lung cancer is the number one cause of cancer-associated mortality[1] with a devastatingly low 5-year survival rate of 16%[2]. The majority of patients present with locally advanced or metastatic disease at the time of diagnosis[2] decreasing their survival rate from 55% to 4% (seer.cancer.gov). Consequently, the survival of these patients becomes dependent on the success of chemotherapeutic and targeted treatment. The PI3K/AKT pathway is an attractive target for NSCLC treatment as genetic alterations are common among its components ultimately promoting PI3K signalling[3]. Inhibitors of the PI3K pathway such as EGFR TKIs and ALK inhibitors have been approved for clinical use, but less than 20% of patients present with one of these mutations[4, 5]. AKT is overexpressed in 50–70% of NSCLC tumors[6] and accordingly, AKT inhibitors MK-2206 and AZD5363 are currently undergoing clinical trials for lung cancer treatment. The data is not yet available for AZD5363, but MK-2206 has completed a phase II clinical trial in combination with erlotinib meeting the pre-determined clinical efficacy in wild-type EGFR patients. However, the results were disappointing with no complete responders[7].

AKT inhibitors have been successful in overcoming resistance to platinum-based chemotherapies as well as EGFR TKIs[8–11], but as a monotherapy, the inhibitors are not producing desirable results[7, 11]. The AKT inhibitors in clinical trials indistinguishably target all three isoforms of AKT. Previously the biological functions of the AKT isoforms were believed to be largely redundant but each isoform has its own unique properties. AKT-1 is important in growth and is ubiquitously expressed across tissues[12, 13]. AKT-2 plays a vital role in glucose homeostasis and is expressed in insulin-responsive tissues[12, 14]. AKT-3 is involved in brain development and is expressed predominantly in the testes and brain[12, 15]. Recent evidence has shown that these isoforms also play distinct roles in lung tumorigenesis. In both a transgenic and viral-oncogene induced mouse model of lung cancer, *Akt1* ablation delayed and decreased tumorigenesis while *Akt2* ablation accelerated and promoted tumorigenesis[16, 17].

To investigate the potential of exclusive AKT-1 inhibition for NSCLC treatment, we compared the effects of an AKT-1 inhibitor A-674563 to the pan-inhibitor MK-2206 on the survival of 6 human NSCLC cells.

## Methods

### Cells

A549, A427, NCI-H23, NCI-H358, NCI-H1975, and NCI-H1650 cells were purchased from American Type Culture Collection. The cells were cultures in RPMI 1640 media supplemented with 10% FBS and 1% antibiotic/antimitotic (ThermoFisher Scientific, Waltham, MA).

### Cell viability assays

Cells were plated in 96 well cell culture plates at a seeding density of 1000 cells/well (A549 cells) or 2000 cells/well (A427, NCI-H23, NCI-H358, NCI-H1975, and NCI-H1650 cells). Cells were incubated overnight at 37° C and 5% CO_2_. They were then treated with DMSO, A-674563 (AKT-1 inhibitor), MK-2206 (pan-AKT inhibitor), PHA-848125 (CDK2 inhibitor), or H-89 2HCl (PKA inhibitor) from Selleck Chemicals (Houston, TX). Media and inhibitor were replaced every 24 hours and survival was measured after 72 hours of treatment. Cells were incubated with 100μL of fresh media and 10μL of WST-1 reagent (Roche Canada, Mississauga, ON) for 2–4 hours. Optical density was determined at 450nm using the EL800 Universal Microplate Reader (BioTek, Winooski, VT) and CalcuSyn software (Biosoft, Cambridge, UK) was used to determine the IC50 concentrations.

### RNA isolation and qRT-PCR

RNA was isolated using the RNeasy Mini Kit (Qiagen Inc, Toronto, ON) according to manufacturer protocol. RNA was reverse transcribed using qScript cDNA mix from Quantabio (Beverly, MA). Gene expression was analyzed by qPCR reactions with SYBR Green qPCR Mastermix (Bioline Reagents Limited, London, ON) and performed on the CFX Connect Real-time PCR Detection system (Bio-Rad Canada, Mississauga, ON). Primers for human *Akt1*, *Akt2*, *Akt3* and *Hprt* were purchased from Bio-Rad Canada (Mississauga, ON). Relative quantification was determined by normalizing expression to *Hprt* using CFX-Manager 3.1 (Bio-Rad Canada, Mississauga, ON).

### Western blotting

Cells were pre-treated for 1 hour in serum-free media and then treated for 30 minutes, 1 hour, and 2 hours. Cells were lysed with RIPA lysis buffer (50mM Tris HCl pH 7.5, 150mM NaCl, 1% Triton X-100, 0.1% sodium dodecyl sulfate, 10mM EDTA, 1% sodium deoxycholate) where each mL was supplemented with 10uL of Phosphatase Inhibitor Cocktail A, B, and C (ThermoFisher Scientific, Waltham, MA). Lysates were left on ice for 20 minutes and then centrifuged at 14,000xg for 20 minutes at 4°C. The supernatant was collected and protein concentration was determined using Bradford assay reagents (Bio-Rad Canada, Mississauga, ON). Reduced protein was separated in 8–12% sodium dodecyl sulphate (SDS)- polyacrylamide gels for 2 hours with 125V using the XCell SureLockTM Mini-Cell Electrophoresis System (ThermoFisher Scientific, Waltham, MA). Protein was transferred at 25V for 1.5 hours onto Hybond ECL nitrocellulose membranes (GE Healthcare Life Sciences, Mississauga, ON) using wet transfer. The membranes were then blocked for 45 minutes in 5% skim milk in tris-buffered saline containing 1% tween (TBST) at room temperature. The membranes were incubated with primary antibodies AKT (1:1000), p-AKT (Ser473) (1:1000), p-AKT (Thr308) (1:500), AKT-1 (1:1000), p-AKT-1 (1:1000), AKT-2 (1:1000), p-AKT-2 (1:1000) (#8599), AKT-3 (1:1000), p-MDM2 (1:1000), p- AS160 (1:1000), p-GSK-3β (1:1000), p-PRAS40 (1:1000), p-S6 ribosomal protein (1:1000), p- FOX01 (1:1000), p-NF-kappaB p65 (1:1000), CDK2 (1:1000), p-CDK2 (1:1000), and β actin (1:5000) from New England Biolabs, Ltd (Whitby, ON) and p-p21 (1:1000) from Santa Cruz Biotechnology (Dallas, TX) diluted in 5% BSA in TBST overnight at 4°C. Proteins were detected with HRP- linked anti-rabbit IgG secondary antibody (1:2000) (New England Biolabs, Ltd, Whitby, ON) and Clarity Western ECL substrate (Bio-Rad Canada, Mississauga, ON). The protein was then imaged using the ChemiDocTMXRS+ System (Bio-Rad Canada, Mississauga, ON) and quantification was performed using Image Lab software (Bio-Rad Canada, Mississauga, ON).

### Flow cytometry

Cells were plated in 100mm culture dishes at 300,000 cells/well (A549 cells) and 450,000 cells/well (A427, NCI-H23, NCI-H358, NCI-H1975, NCI-H1650 cells) and incubated overnight. The cells were then treated with the same concentration of A-674563 and MK-2206 (IC50 for A-674563) or a DMSO control. After 24 hours of treatment, the FITC BrdU Flow Kit (BD Biosciences, Mississauga, ON) was used to determine the cell cycle profiles of the treated cells. Fluorescence was measured by the BD Accuri C6 flow cytometer (BD Biosciences, Mississauga, ON) at a rate of less than 400 events/second. Analysis was performed with Accuri C6 software (BD Biosciences, Mississauga, ON).

### Statistical analysis

Statistical Analysis was performed using Graphpad Prism 6 (GraphPad Software, Inc., La Jolla, CA). Comparison of two means at multiple concentrations was determined with multiple T-tests using the Holm-Sidak method. Comparison of multiple means was executed using one-way ANOVA followed by a post-hoc Tukey’s Test. Comparison of multiple means split into multiple variables was determined with two-way ANOVA followed by a post-hoc Tukey’s Test. Error is represented by Standard Error of the Mean (SEM) or Standard Deviation (SD). Statistical significance is noted as p<0.05.

## Results

### A-674563 reduces NSCLC cell proliferation more effectively than MK-2206

**Fig 1:**
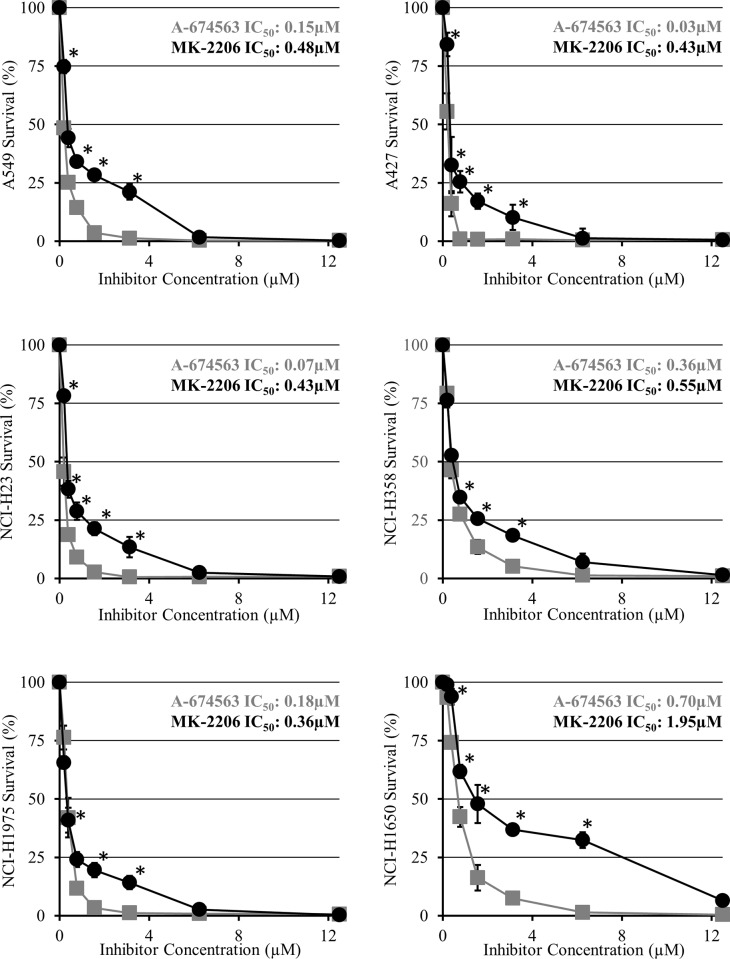
Cell survival curves of A) A549, B) A427, C) NCI-H23, D) NCI-H358, E) NCI-H1975, F) NCI-H1650 cells treated with increasing doses of A-674563 and MK-2206 for 72 hours. Cells were incubated with cell proliferation reagent WST-1 for 2 hours and absorbance was read at 450nm. Optical density was then normalized to a 1% DMSO control. The data is presented as the percentage of cell survival relative to the DMSO control ± SEM of three independent trials. Statistical significance was determined with multiple T-tests using the Holm-Sidak method without assuming a consistent SD and is represented by *p<0.05.

To compare the effects of AKT-1 and pan-AKT inhibition on human NSCLC cell proliferation, six NSCLC cell lines were treated with increasing doses of both A-674563 and MK-2206 over a 72-hour period. A-674563 significantly decreased NSCLC cell proliferation to a greater extent than MK-2206 and had a lower IC_50_ value in all 6 cell lines (Fig 1). The difference in efficacy between the two inhibitors was most prominent in the cell lines harbouring *Stk11* mutations, the A549, A427, and NCI-H23 cells. The improved potency of A-674563 over MK-2206 was also confirmed with manual cell counts performed after treatment with both MK-2206 and A-674563 for 72 hours at the IC_50_ values determined for A-674563.

**Fig 2:**
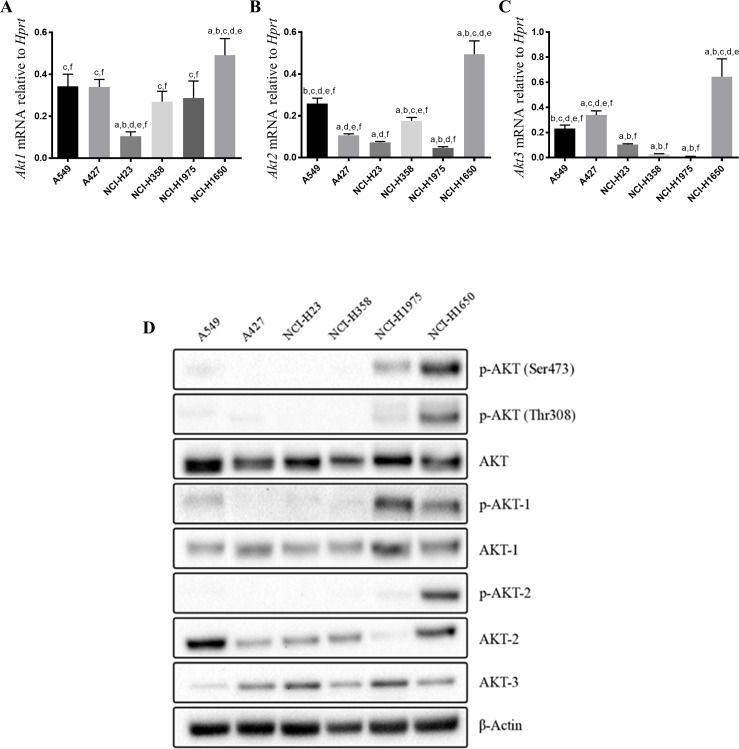
A) Basal mRNA expression levels of A) *Akt1*, B) *Akt2* and C) *Akt3* in six NSCLC cell lines presented as the Mean ± SD with letters representing groups that are statistically different from one another such that (a) represents A549 cells, (b) A427 cells, (c) NCI-H23 cells, (d) NCI-H358 cells, (e) NCI-H1975 cells, and (f) NCI-H1650 cells. Samples were normalized to an *Hprt* housekeeping gene and statistical significance (p<0.05) was determined with one-way ANOVA and Tukey’s Multiple Comparison Tests. Results are based on 3 biological replicates and two technical replicates. D) Representative western blot of the basal protein levels of total AKT, phosphorylated AKT at serine 473 or threonine 308, total AKT1, AKT2 and AKT3 as well as the phosphorylated forms of AKT1 and AKT2 in six NSCLC cell lines. Phosphorylated AKT3 was not evaluated as no specific antibody for phosphorylated AKT3 is currently available. β-actin served as a loading control.

To explain the difference in efficacy of the inhibitors across the cell lines we measured the basal mRNA (Fig 2) and protein levels of AKT-1, AKT-2, and AKT-3 (Fig 2). We found that the mRNA levels were not good predictors of the protein levels. In addition, there was no expression patterns to explain the difference in sensitivity of the cell lines to the inhibitors and between the inhibitors. However, it was confirmed that all 3 AKT isoforms are expressed in each of the cell lines.

### A-674563 and MK-2206 diverge in their effects on cell cycle progression but not apoptosis

**Fig 3:**
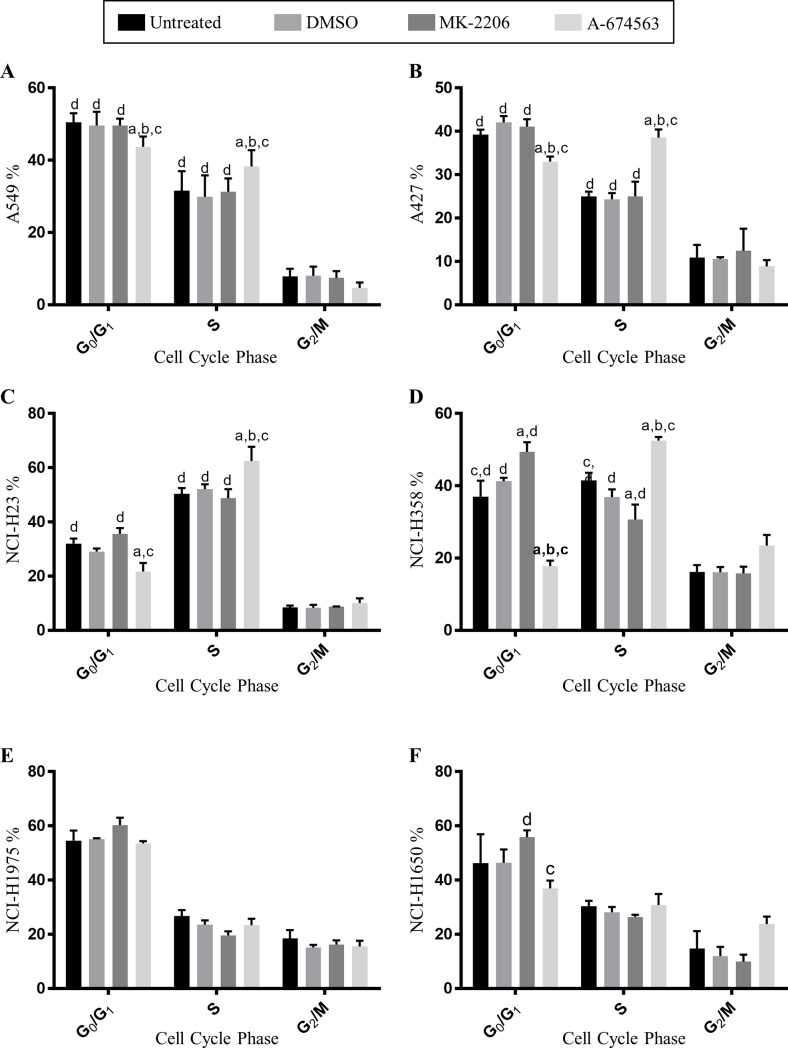
Cell cycle analysis of A) A549, B) A427, C) NCI-H23, D) NCI-H358, E) NCI-H1975 and F) NCI-H1650 cells treated over a 24-hour period treated with A) 0.15μM, B) 0.03μM, C) 0.07μM, D) 0.36μM, E) 0.18μM and F) 0.70μM of both A-674563 and MK-2206. Data is presented as the Mean ± SEM with letters representing groups that are statistically different from one another such that (a) represents untreated cells, (b) DMSO treated cells, (c) MK-2206 treated cells, and (d) A-674563 treated cells. Statistical significance (p<0.05) was determined with two-way ANOVA and Tukey’s Multiple Comparison Tests. Results are based on 3 biological replicates.

To determine the effects of MK-2206 and A-674563 on cell cycle progression, BrdU flow cytometry was performed after 24 hours of treatment. In the *Stk11* mutant A549, A427, and NCI-H23 cells, A-674563 treatment significantly altered cell cycle progression compared to the controls. A-674563 decreased the proportion of cells in the G0/G1 phase, significantly increased the proportion of cells in the S phase, with no significant changes in the G2/M phase (Fig 3). On the contrary, treatment with MK-2206 induced no significant changes in these cell lines. In the NCI-H358 cells, A-674563 also significantly decreased the proportion of cells in the G0/G1 phase, significantly increased the proportion in the S phase, with no significant changes in the G2/M phase (Fig 3). But unlike the other cell lines, MK-2206 treatment significantly increased the proportion of cells in the G0/G1 phase and significantly decreased the proportion of cells in the S phase with no changes in the G2/M phase (Fig 3). Intriguingly, the NCI-H358 cells were found to have smallest difference in efficacy between A-674563 and MK-2206 compared to the other 5 cell lines (Fig 1). In the NCI-H1975 cells, neither inhibitor induced any significant changes and in the NCI-H1650 cells, the only significant difference was between A-674563 and MK-2206 treatment for the G0/G1 phase of the cell cycle (Fig 3). We also investigated the effects of the inhibitors on apoptosis but there were no significant differences between the inhibitors or the controls.

### p-AKT levels are decreased by MK-2206 and increased by A-674563

**Fig 4:**
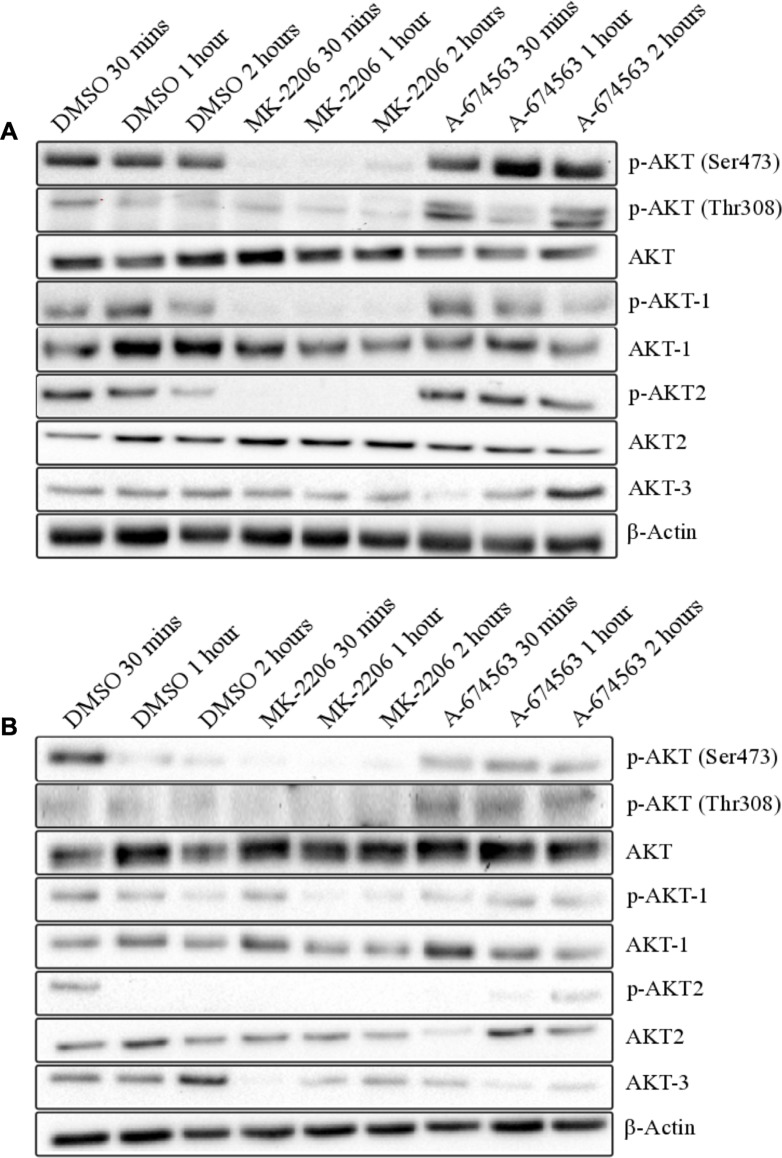
Representative western blot results of A) A549 and B) A427 cells after treatment with MK-2206 and A-674563 at the IC_50_ determined for the A-674563 inhibitor. Cells were pre-treated for 1 hour in serum-free media and protein was extracted after 30 minutes, 1 hour, and 2 hours of treatment in serum-containing media. Results are based on two biological replicates.

Western blots were performed on the A549 and A427 cells to measure the protein levels of the total and active AKT isoforms following treatment with A-674563 and MK-2206 (Fig 4). A-674563 was found to increase the expression of p-AKT (Ser473) and p-AKT (Thr308) in both cell lines. A-674563 also increased p-AKT-1 and p-AKT-2 expression in the A549 cells and increased expression over time in the A427 cells. MK-2206 treatment decreased the expression of p-AKT (Ser473), p-AKT (Thr308), p-AKT-1, and p-AKT-2 in both the A549 and A427 cells.

### Protein expression of AKT targets did not reveal the mechanism for the improved efficacy of A-674563

**Fig 5:**
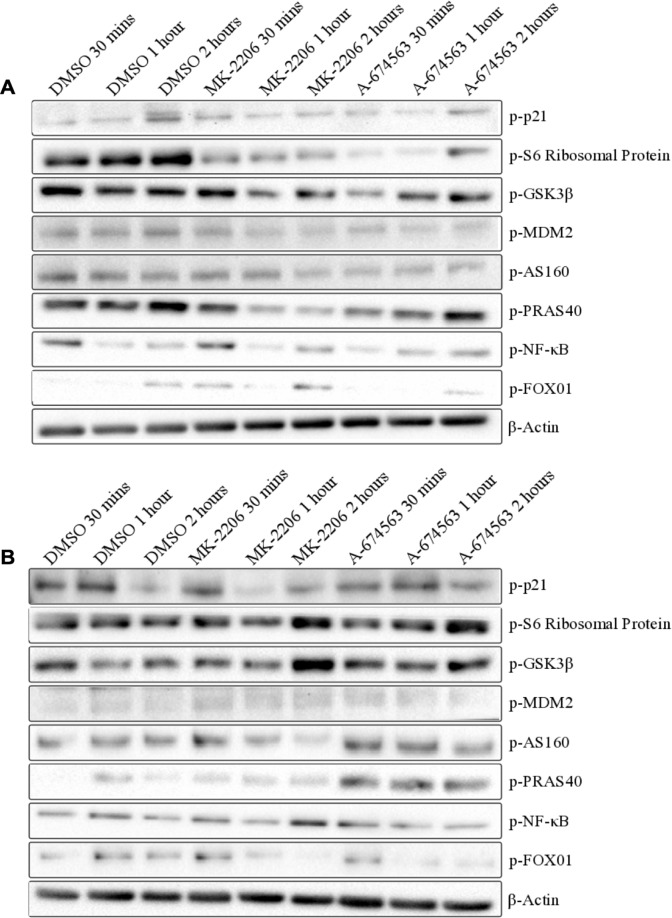
Representative western blot results of A) A549 and B) A427 cells after treatment with MK-2206 and A-674563 at the IC_50_ determined for the A-674563 inhibitor. Cells were pre-treated for 1 hour in serum-free media and protein was extracted after 30 minutes, 1 hour, and 2 hours of treatment in serum-containing media. Results are based on two biological replicates.

To determine whether A-674563 differentially regulated signaling downstream of AKT compared to MK-2206, protein levels of 8 potential AKT targets were measured after treatment with MK-2206 and A-674563 in A549 (Fig 5) and A427 cells (Fig 5). Protein was extracted after 30 minutes, 1 hour, and 2 hours of treatment. Both A-674563 and MK-2206 had similar effects on p-p21, p-S6 ribosomal protein, and p-GSK-3β expression, and thus these targets presumably do not contribute to the observed differences in cell growth/survival between A-674563 and MK-2206. p-NF-κB and p-FOX01 expression were differentially impacted by the inhibitors both within and between the two cell lines so they do no account for the benefits of A-674563 either. However, p-MDM2 and p-AS160 were similarly effected by both inhibitors in the A549 but not the A427 cells. Expression was decreased by both inhibitors in the A549 cells but increased by A-674563 in the A427 cells. p-AS160 expression was decreased by both inhibitors in the A549 cells and in the A427 cells, MK-2206 decreased expression and A-674563 increased expression at all the time points. p-PRAS40 expression was also impacted by A-674563 treatment inconsistently between the A549 and A427 cells. Where expression was unchanged by A-674563 in the A549 cells but markedly increased in the A427 cells. Since there was a dramatic difference in the effect of A-674563 between the two cell lines, it’s possible that increased p-MDM2, p-AS160, and p-PRAS40 expression either contribute to or result from the increased sensitivity of the A427 cells. However, none of the 8 substrates were oppositely effected by the two inhibitors in both the cell lines. Therefore, we were unable to pinpoint exactly which part of the pathway is responsible for the benefits of A-674563 over MK-2206.

### Off-target CDK2 inhibition contributes to the potency of A-674563

**Fig 6:**
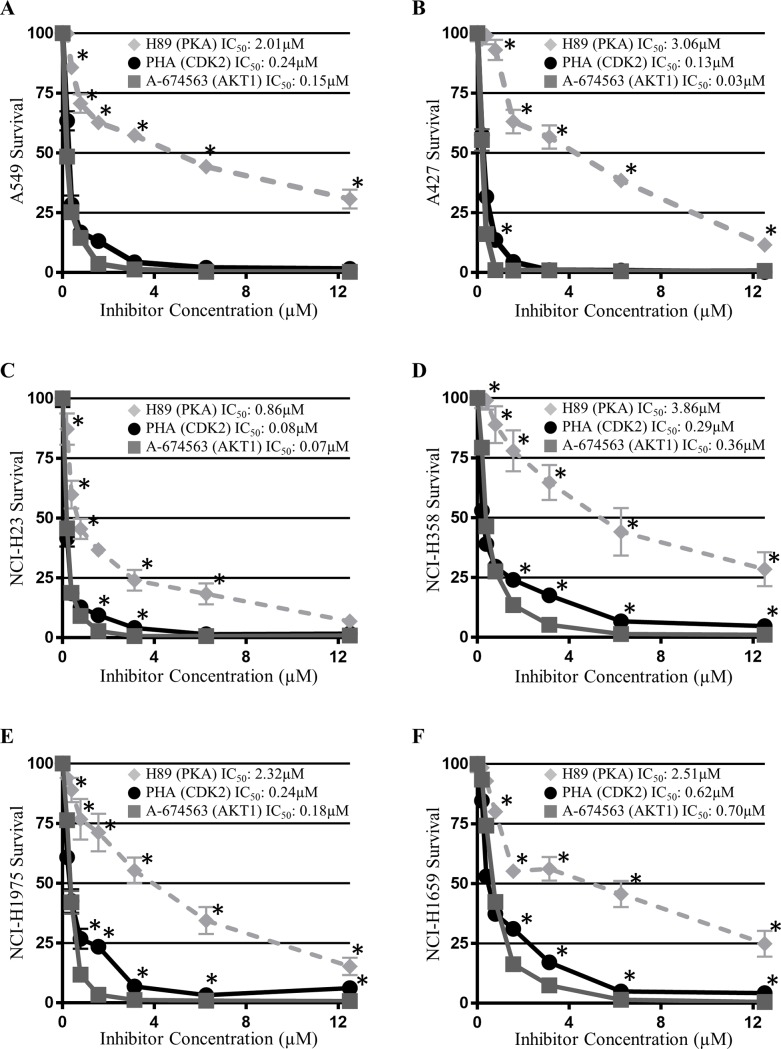
Cell survival curves of A) A549, B) A427, C) NCI-H23, D) NCI-H358, E) NCI-H1975, and F) NCI-H1650 cells treated with increasing doses of A-674563, PHA-84812, and H89 2HCl for 72 hours. Cells were incubated with cell proliferation reagent WST-1 for 2 hours and absorbance was read at 450nm. Optical density was then normalized to a 1% DMSO control. The data is presented as the percentage of cell survival relative to the DMSO control ± SEM of three independent trials. Statistical significance was determined with multiple T-tests using the Holm-Sidak method without assuming a consistent SD and is represented by *p<0.05.

**Fig 7:**
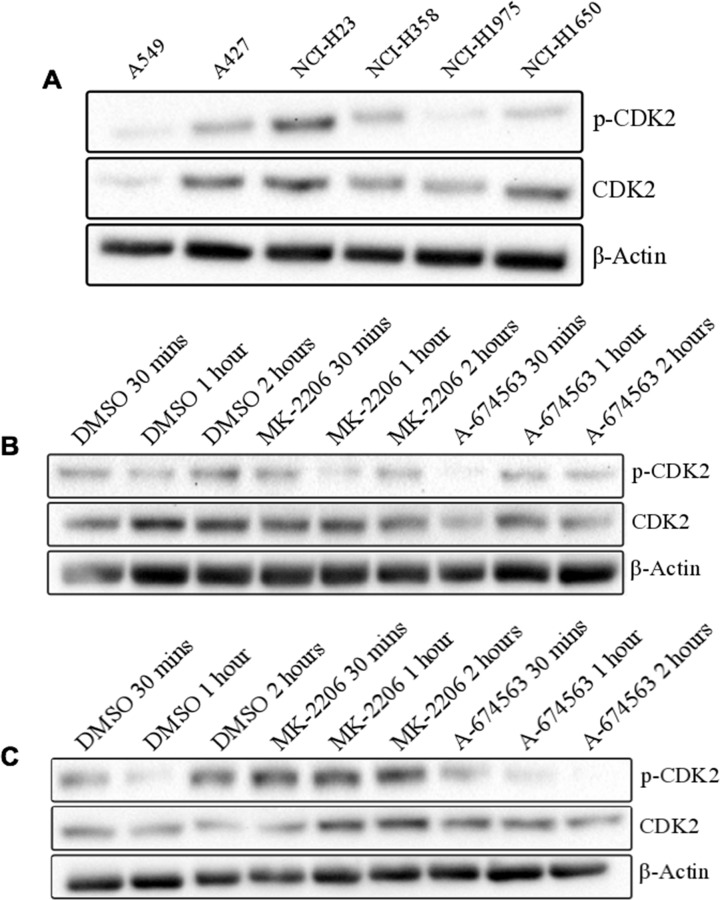
A) Basal protein levels of p-CDK2 and CDK2 across six NSCLC cell lines. Protein expression levels of CDK2 and p-CDK2 in B) A549 and B A427 cells after treatment with MK-2206 and A-674563 at the IC_50_ determined for A-674563. Cells were pre-treated for 1 hour in serum-free media and protein was extracted after 30 minutes, 1 hour, and 2 hours of treatment in serum-containing media. Results are based on two biological replicates.

Since western blots were unable to explain the improved efficacy of A-674563 over MK-2206, we evaluated the contribution of the off-target effects of A-674563 on its observed potency. A-674563 is known to inhibit both PKA and CDK2. We performed a dose escalation study on the six NSCLC cell lines with a PKA inhibitor H89 2HCL and a CDK2 inhibitor PHA-84812 to evaluate their effects on cell proliferation (Fig 6). The WST-1 assays revealed that the reduction in cell proliferation resulting from CDK2 inhibition largely mirrored the reduction observed with A-674563 treatment. In addition, the determined IC_50_ values for the CDK2 inhibitor PHA-84812 were similar to those measured for A-674563 in each of the cell lines. Basal protein expression levels of CDK2 and p-CDK2 were measured across the 6 cell lines (Fig 7). CDK2 and p-CDK2 expression were highest in the A427, NCI-H23, and NCI-H1650 cells. Notably, between each of the mutation pairs (*Kras* mutants, *Kras/p53* mutants, *p53/EGFR* mutants), A427, NCI-H23 and NCI-H1650 cells are marginally more sensitive to A-674563 compared to MK-2206. Western blots were also performed on the A549 and A427 cells to confirm that treatment with A-674563 inhibits CDK2 (Fig 7). It was observed that A-674563 deceased p-CDK2 expression initially in the A549 cells (Fig 7) and at all time points in the A427 cells (Fig 7) while MK-2206 had less dramatic effects on p-CDK2 levels. All together, these findings suggest that CDK2 inhibition likely plays a pivotal role in the efficacy of A-674563.

## Discussion

AKT is a key regulator of cellular growth, proliferation, and survival making it a prominent therapeutic target in cancer research. Active AKT is commonly overexpressed in NSCLC[18–20] and inhibition of AKT helps to overcome chemotherapeutic resistance[9, 10]. Accordingly, AKT inhibition is under clinical investigation for lung cancer treatment. Thus far, results have been disappointing with the pan-AKT inhibitor MK-2206 rendering no complete responders in combination with erlotinib [7]. However, selective AKT inhibition may serve to be a more viable treatment option as the AKT isoforms have demonstrated to play opposing roles in lung tumorigenesis[16].

In this investigation, we showed that the AKT-1 inhibitor A-674563 more potently reduced NSCLC cell proliferation than the pan- AKT inhibitor MK-2206. In all six NSCLC cell lines, A-674563 had a lower IC50 value than MK-2206 with the largest difference in efficacy among the *Stk11* mutant cell lines (A549, A427 and NCI-H23 cells). These three cell lines all have a genetic aberration in the *Stk11* gene encoding liver kinase B 1 (LKB1), a tumor suppressor protein that prevents the G1 to S phase transition[21]. Excluding the p53/EGFR mutant cell lines, A-674563 induced significant cell cycle changes in all of the cell lines while MK-2206 only induced significant changes in the *Stk11* wild-type NCI-H358 cells. In this cell line, MK-2206 was observed to impair the G1 to S phase transition which corresponds to the function of LKB1. This indicates that *Stk11* mutant cell lines may be resistant to MK-2206 which is synonymous to what was reported by Byers et al. MK-2206 inhibits AKT activity which in turn prevents the phosphorylation and inhibition of LKB1[22]. Accordingly, MK-2206 may alter cell cycle progression by attenuating AKT-induced inhibition of LKB1. In this respect, since the *Stk11* mutant cell lines lack functional LKB1 protein normal LKB1 function cannot be restored even in the absence of AKT suppression.

The significant cell cycle changes induced by A-674563 include a decrease in the proportion of cells in the G0/G1 phase and an increase in the proportion of cells in the S phase. Since there are more cells undergoing DNA synthesis, it’s anticipated that there would be an increase in the percentage of cells undergoing mitosis, but no significant change was found. This indicates that A-674563 is preventing cells from progressing to the G2/M phase of the cell cycle. A-674563 is known to have an off-target effect of inhibiting CDK2 activity. The cell cycle profile resulting from A-674563 treatment is congruent with the findings of Zhu et al.[23] who reported that CDK2 inhibition increases the proportion of cells in the S phase by inducing DNA re-replication. DNA re-replication is a novel mechanism for cancer therapy currently being investigated in a phase II clinical trial for leukemia. In this study, patients are being treated with MLN4924 an inhibitor of NEDD8-activating enzyme. MLN4924 induces apoptosis, autophagy, cellular senescence[24], and decreases cellular proliferation by inducing DNA re-replication and subsequently activating the DNA damage checkpoint pathway[25]. In accordance with these findings, it’s possible that one of the major drivers for the improved efficacy of A-674563 over MK-2206 could be due to its induction of cellular re-replication through CDK2 inhibition.

Given the observed differences in cell cycle progression, it's anticipated that the effects of the inhibitors would deviate at some point in the AKT pathway. However, no obvious differences in protein expression were identified among the downstream targets. There were no opposing effects of the inhibitors that were present in both of the cell lines and therefore we cannot define the divergent effects of A-674563 and MK-2206 on the downstream targets of AKT. We only measured the expression of 8 out of 254 of AKTs known downstream targets so its possible that the effectors chosen are not implicated in the varied efficacies of the two inhibitors. Although we were unable to identify divergent effects of the inhibitors on protein expression, heterogenous effects of A-674563 were present between the A549 and A427 cells. The two cell lines share a similar mutation profile but A-674563 is drastically more potent than MK-2206 in the A427 cells (14.9x difference in IC50 value) compared to the A549 cells (3.2x difference in IC50 value). Accordingly, other factors in addition to *Stk11* mutations dictate a cell lines sensitivity to A-674563 treatment. Between the A549 and A427 cells, there was a variation in the effects of A-674563 on the phosphorylation of the downstream effector PRAS40. PRAS40 is known to be an inhibitor of mTORC1 and direct phosphorylation of PRAS40 by AKT alleviates this effect[26, 27]. Regardless, numerous studies have published conflicting data suggesting that loss of PRAS40 activity may inhibit mTORC1[28–31]. In our western blots, treatment with A-674563 in the A549 cells initially decreased and then maintained expression of p-PRAS40 the same as the controls. In the A427 cells, A-674563 markedly increased expression above the controls at all time points. Considering the controversial reports in the literature, it’s possible that this increase in p-PRAS40 expression may actually be advantageous in the efficacy of the inhibitor or may just be a result of feedback within the PI3K/AKT pathway.

The effect of A-674563 on the expression of p-AS160 and p-MDM2 also varies between the A549 and A427 cells. A-674563 treatment decreases the expression of p-AS160 and p-MDM2 in the A549 cells and increases the expression in the A427 cells. Intriguingly, both of these kinases are potential AKT-2 specific targets[32, 33]. Compensation is a common occurrence when targeting the individual AKT isoforms as double knockouts result in lethality while single AKT isoform null mice are viable[34]. Moreover, loss of either AKT-1 or AKT-2 does not change total phosphorylated AKT levels[35]. Since A-674563 targets solely AKT-1, at least some compensation by AKT-2 is expected. Treatment with A-674563 increases p-AKT-2 expression in both the A427 and A549 cells but this increase is similar to that observed for p-AKT and p-AKT-1 with A-674563 treatment. As a result, we cannot assume that this increase reflects AKT-2 activity. However, A-674563 decreases expression of p-MDM2 and p-AS160 in A549 cells and increases expression in the A427 cells. Thus, A-674563 may only be increasing AKT-2 activity in the A427 cells. Potentiated AKT-2 activity is presumed to be advantageous in the efficacy of A-674563 since AKT-2 ablation promotes and accelerates tumorigenesis in transgenic mouse models[16, 17]. It’s possible that we’re only observing this compensation effect in the A427 cells due to basal AKT-2 expression levels since we found A549 cells to have higher AKT-2 protein and mRNA expression.

In summary, exclusive inhibition of AKT-1 has proven to be a more effective strategy for in vitro NSCLC treatment than pan-AKT inhibition. CDK2 inhibition also appears to play a vital role in the efficacy of A-674563 supporting further investigation as a therapeutic target.
